# Supplementary material for: Decoding past microbial life and antibiotic resistance in İnonü Cave’s archaeological soil
Source: PLoS One. 2025 Jul 31;20(7):e0326358. doi: 10.1371/journal.pone.0326358 (PMC12312911; doi:10.1371/journal.pone.0326358)
Supplement: S1 File — (PDF) [file pone.0326358.s001.pdf]

Supporting Information

Decoding Past Microbial Life and Antibiotic Resistance in İnonü Cave's Archaeological Soil

Short Title: "Integrating Archaeology and Microbiology: Insights into Ancient Resistance Genes

Sukran Ozturk*[1¶] F. Gülden Ekmen [2¶] Hamza Ekmen[2] Esra Mine Ünal[3,4] Ayşegül Er[3,4] Emre Keskin[3,4,5] Benjamin Stanley Arbuckle[6]

[1:] *Zonguldak Bülent Ecevit University Faculty of Pharmacy Pharmaceutical Microbiology, Zonguldak, Turkiye*

[2:] *Zonguldak Bülent Ecevit University, Faculty of Humanities and Social Sciences, Department of Archeology, Zonguldak, Turkiye*

[3:] *Ankara University, Agricultural Faculty, Department of Fisheries and Aquaculture, Evolutionary Genetics Laboratory (AGL), Ankara, Turkiye*

[4:] *Agrigenomics Hub (AgriGx) Animal and Plant Genomics Research Innovation Centre, Ankara, Turkiye*

[5:] *Ankara University Aquaculture Research and Application Center (ASAUM), Ankara, Turkiye*

[6:] *University of North Carolina at Chapel Hill, Department of Anthropology, USA*

Fig 7: Agarose gel electrophoresis image of PCR results for antibiotic resistance genes in soil samples from Inonu Cave

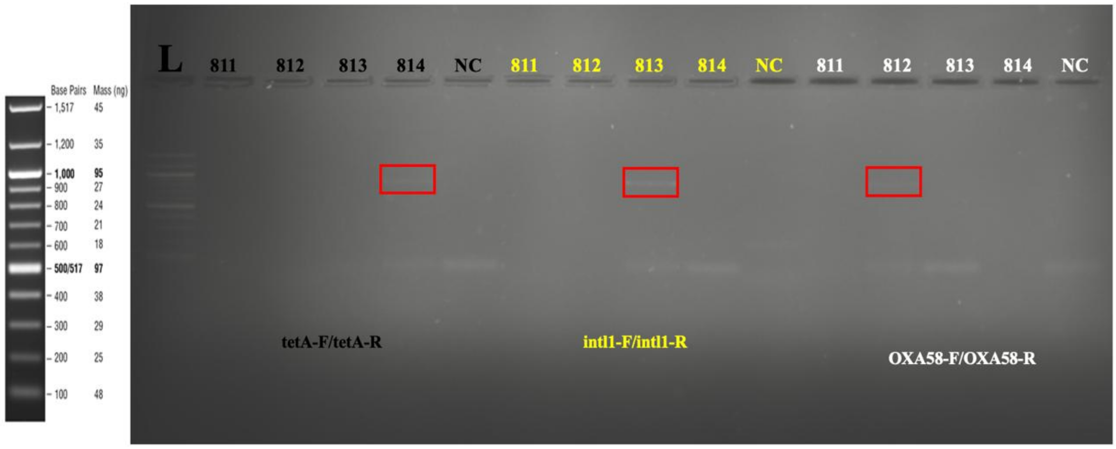

Fig S1: L: Ladder, NC: Negative Control, Chalcolithic Age (sample 814), Bronze Age (sample 813), Late Bronze Age (sample 812), Early Iron Age (sample 811), Primer: tetA-F/tetA-R, intl1-F/intl1-R, OXA58-F/OXA58-R

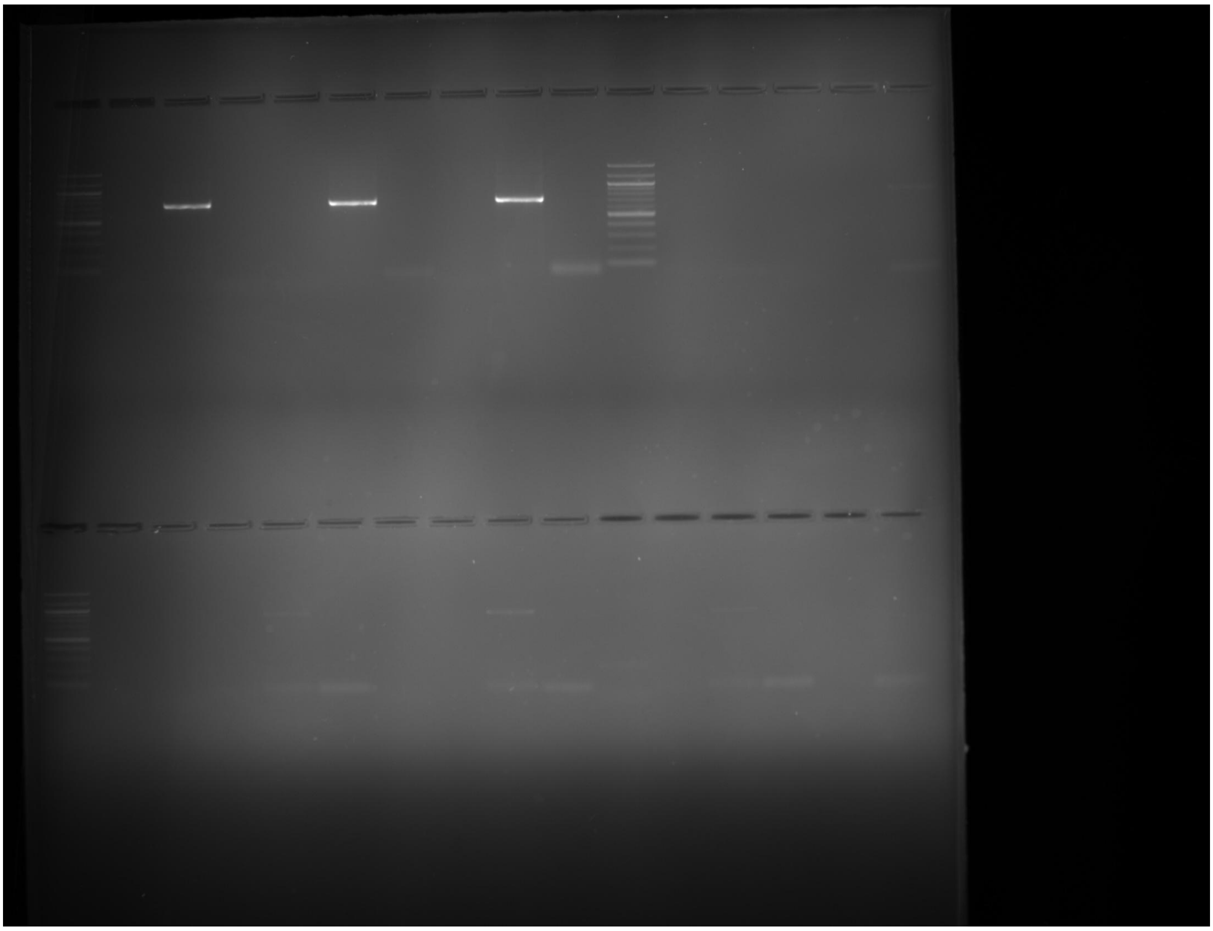

Fig S1: *Raw Jel/blot Agarose gel electrophoresis image of PCR results for antibiotic resistance genes in soil samples from Inonu Cave*

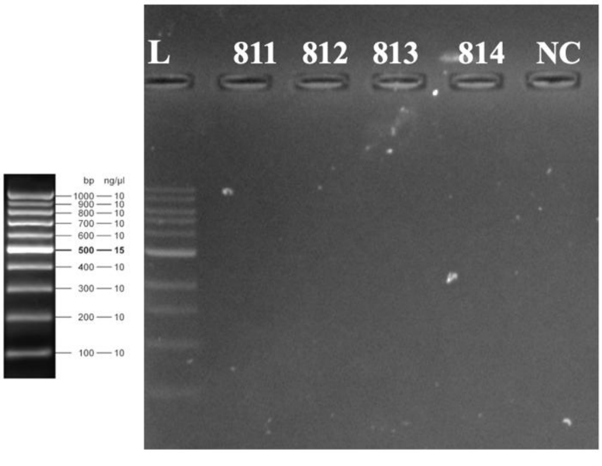

Fig S 2: L: Ladder, NC: Negative Control, Chalcolithic Age (sample 814), Bronze Age (sample 813), Late Bronze Age (sample 812), Early Iron Age (sample 811), Primer: blaTEM-F/blaTEM-R

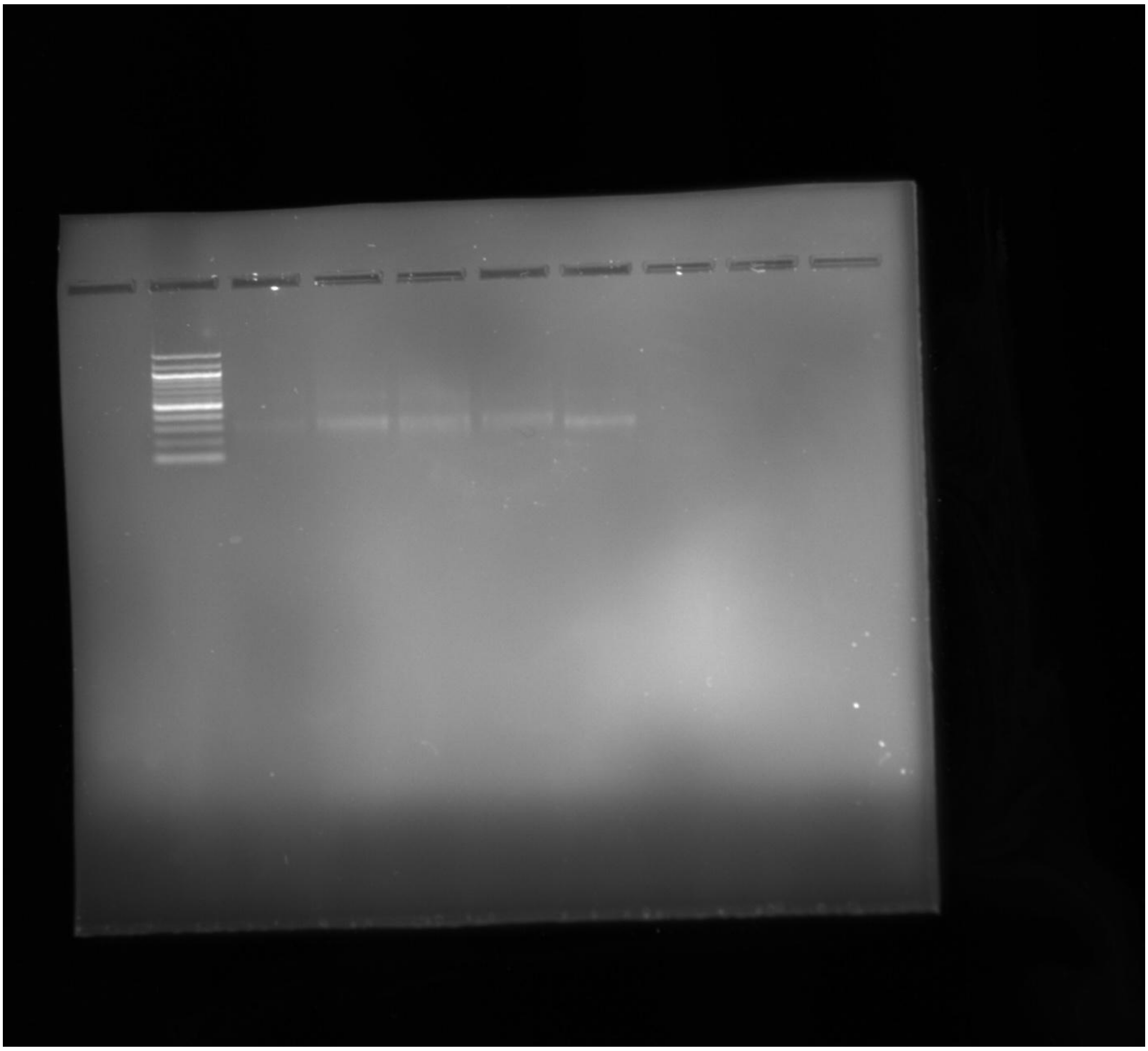

Fig S2: *Raw Jel/blot Agarose gel electrophoresis image of PCR results for antibiotic resistance genes in soil samples from Inonu Cave*

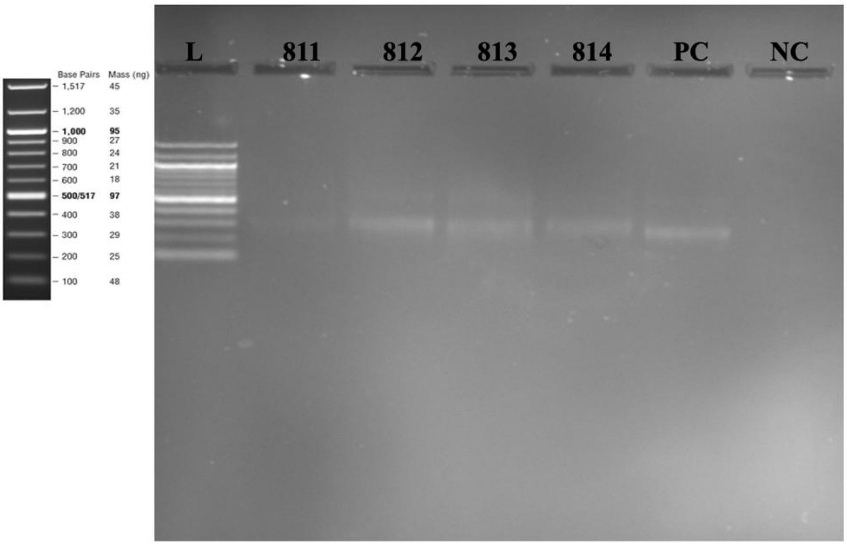

Fig S 3: L: Ladder, NC: Negative Control, PC: Positive Control, Chalcolithic Age (sample 814), Bronze Age (sample 813), Late Bronze Age (sample 812), Early Iron Age (sample 811), Primer: 16SV3-F/16SV3-R

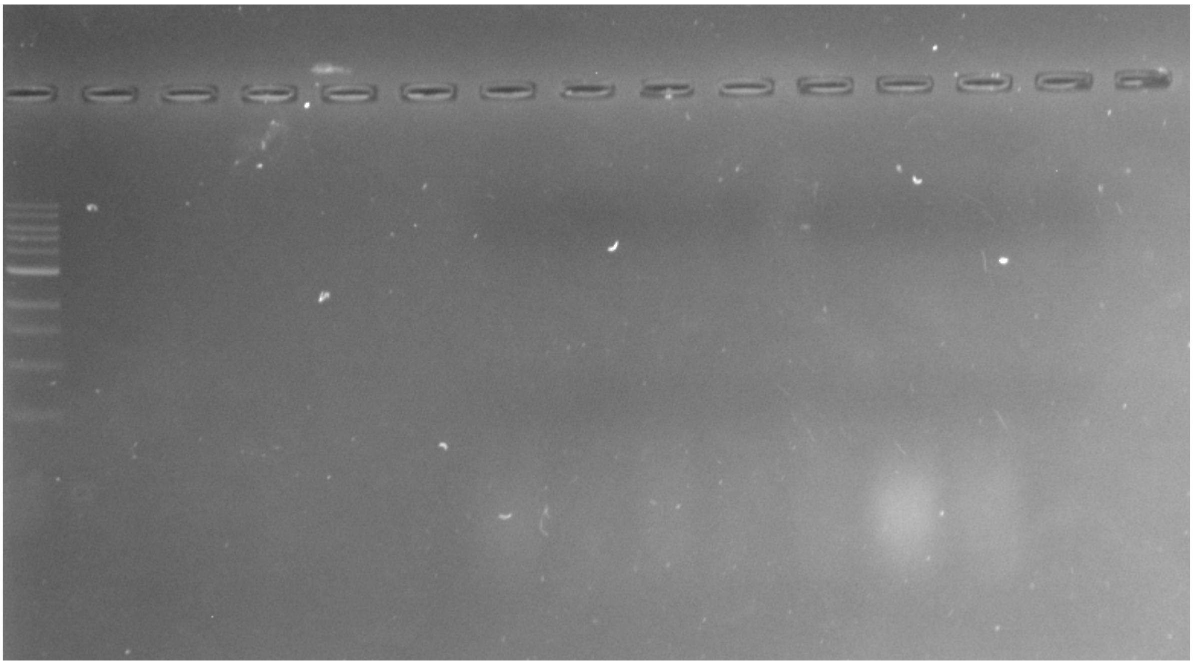

Fig S3: *Raw Jel/blot Agarose gel electrophoresis image of PCR results for antibiotic resistance genes in soil samples from Inonu Cave*
