# Supplementary figures and images for: Decoding past microbial life and antibiotic resistance in İnonü Cave’s archaeological soil
Source: PLoS One. 2025 Jul 31;20(7):e0326358. doi: 10.1371/journal.pone.0326358 (PMC12312911; doi:10.1371/journal.pone.0326358)

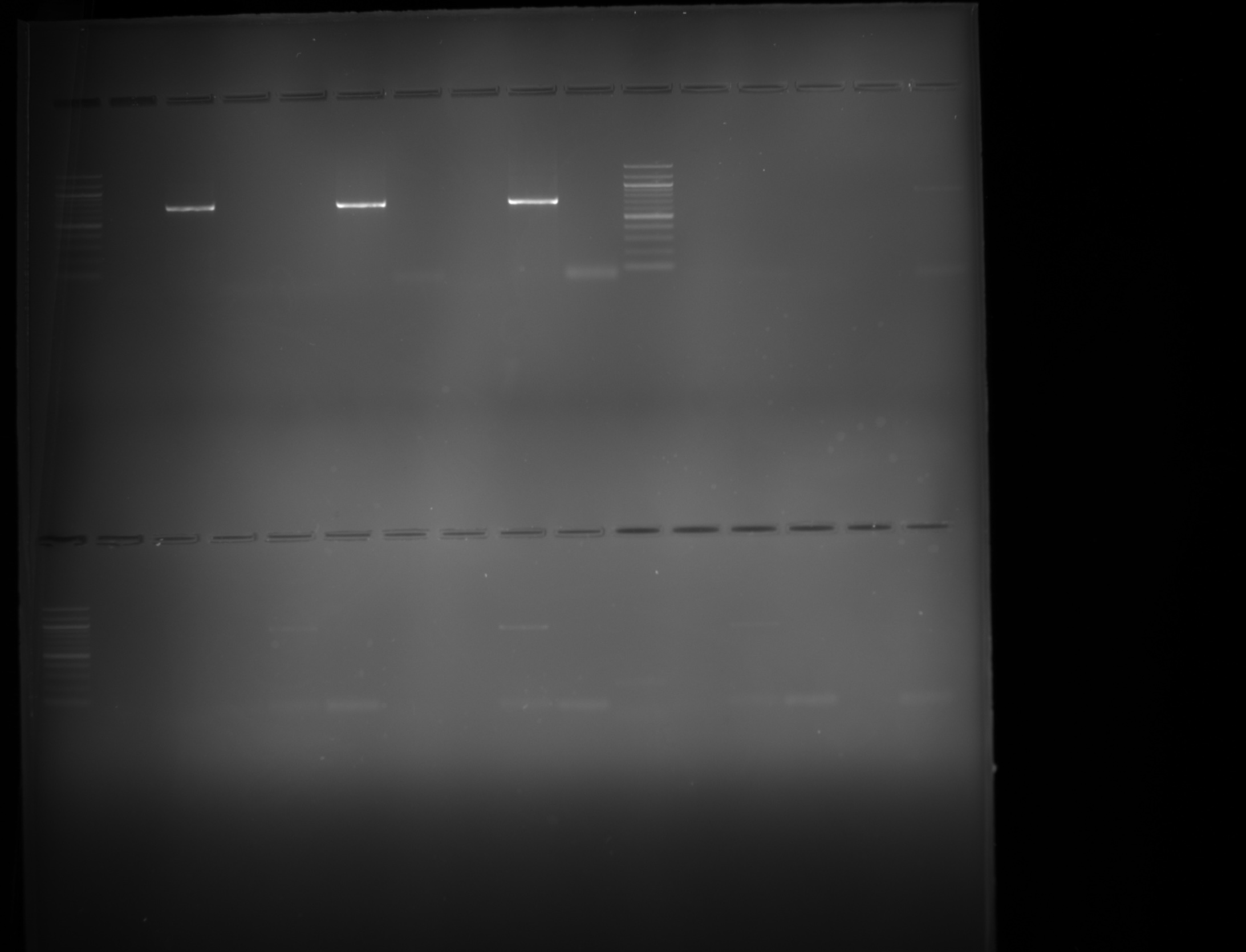

Supplement: S1 Fig — (JPG) [file pone.0326358.s002.jpg]

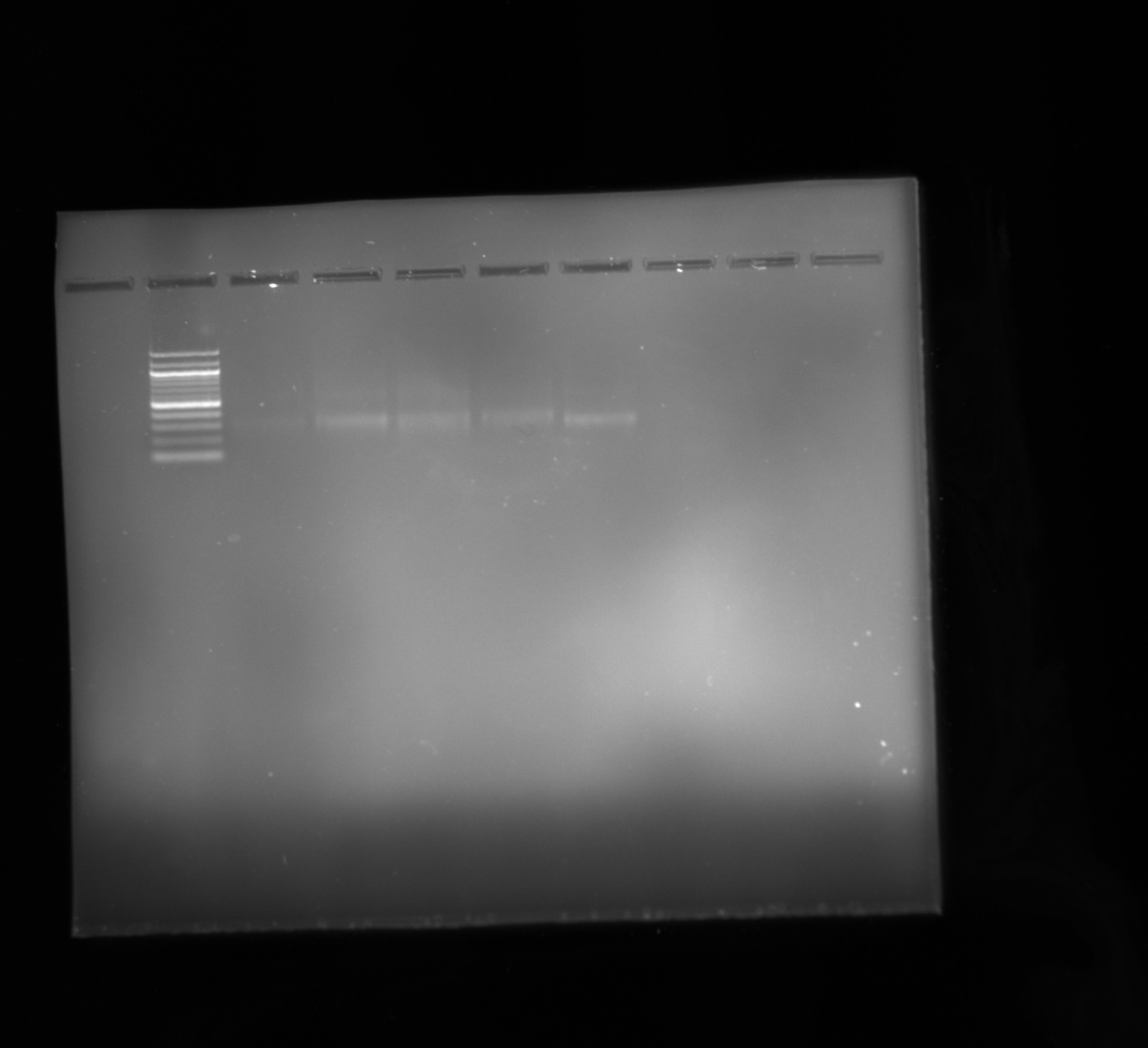

Supplement: S2 Fig — (JPG) [file pone.0326358.s003.jpg]

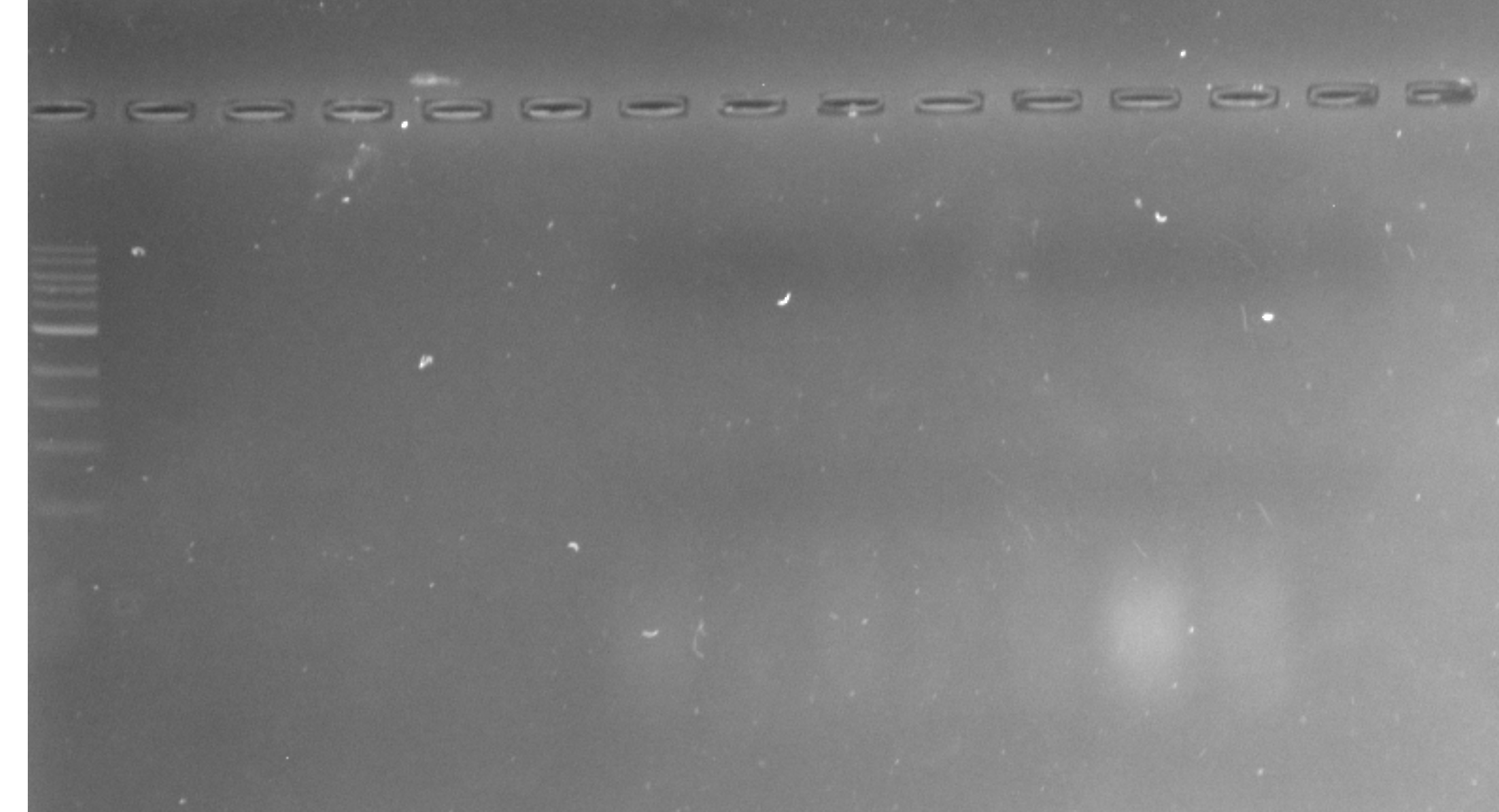

Supplement: S3 Fig — (JPG) [file pone.0326358.s004.jpg]
