# Supplementary material for: Decoding past microbial life and antibiotic resistance in İnonü Cave’s archaeological soil
Source: PLoS One. 2025 Jul 31;20(7):e0326358. doi: 10.1371/journal.pone.0326358 (PMC12312911; doi:10.1371/journal.pone.0326358)
Supplement: Attachment — Submitted filename: renamed_b31ce.pdf [file pone.0326358.s007.pdf]

Revision Decoding Past Microbial Life and Antibiotic Resistance in İnonü Cave's Archaeological Soil

To begin with we would like to thank the authors for addressing our comments and for the overall extensive revision of their manuscript. We appreciate the added archeological details on the site as well as the technical information in the methods section.
However, we would like encourage a few improvement in the following sections:

Introduction

The introduction was well expanded and in its current state adds a lot more relevant context to the study. We would like to highlight again that every statement (especially referring to previous studies) requires a reference. Please revise the text with this in mind. Here are a few examples:

The field of ancient DNA (aDNA) and microbial communities in archaeological contexts has received considerable attention in recent years. These investigations offer a distinct viewpoint on human behavior, health, and interactions with the environment in the past. By examining microbial DNA found in archeological sites, scientists can extract significant insights regarding the microbiomes of ancient civilizations, their food patterns, and the occurrence of contagious Illnesses **(REF)**. Furthermore, examining ancient microbial communities allows us to comprehend the historical influence of human actions on environmental microbial ecosystems. Recent interdisciplinary studies have shown that the materials contained in archaeological sediments provide information beyond just defining culture **(REF)**. In this context, organic remains dating back thousands of years play an important role. While research on organic remains found in archaeological sites has gained momentum in recent years, the number of studies, especially on microbial diversity and change, still needs to be increased. High-throughput sequencing technologies have opened new frontiers in microbial community analysis and have become widely used in assessing the diversity of bacterial components in soil **(REF)**. The recent application of next-generation sequencing methods such as Illumina and Roche provides reliable and accurate results in detecting microbial taxa. 16S rRNA studies are preferred as an excellent phylogenetic method to obtain important information about investigating and detecting microbial taxa and antibiotic resistance genes in soil samples from archaeological excavations **(REF)**.
Now, more than three decades later, scientists and researchers are actively engaged in ongoing research to solve various puzzles related to the origin of humanity, migration patterns, the emergence of infectious diseases, and their spread among ancient populations. aDNA analysis has emerged as a state-of-the-art genetic tool gaining momentum worldwide, changing our comprehensive understanding of the past. For example, in a recent groundbreaking study, aDNA analysis of seven individuals revealed the origin of the plague strain that caused the Black Death in present-day (2) (3). **Are you sure (2) is the appropriate reference here?**
Today, with the help of aDNA, the current atlas of genetic diversity is no longer limited to a glimpse of the diversity observed in modern populations across the globe. Instead, it is continuously updated with datasets that track changes in the genetic origins of human, animal, plant, and even microbial populations as they grow, implode, and adapt to new environmental

factors (4) (5) (6). Beyond its biological and archaeological prospects, aDNA also holds the potential to establish strong and intriguing political and cultural links with other nations (7). More broadly, aDNA data have revolutionized our knowledge and curiosity and have led to the publication of an enormous amount of literature. The latest technological innovations **(If you mention innovations, make sure to name them!** have proven to be an extraordinary tool for scientists and researchers in the race to obtain the 'gold' (aDNA) **I would paraphrase this or leave it out as it is neither common nor scientific to refer to aDNA as "gold".**. In the present research, we aim to broaden the scope of the ongoing conversation in
the field of aDNA by reporting relevant literature published worldwide and detailing advances and challenges.**(Put aims at the end of the introduction rather than in the middle. Further, it seems obsolete to mention you're including a state of the art literature review as this is just common scientific practice in a paper. I would remove this sentence.)** aDNA from microorganisms, including pathogens and commensals, can provide insight into human health and changes in the diet and ecology of diseases.

While significant microbial abundance and diversity were found in soil samples from four archaeological levels, our study does not reveal differences in microbial community structure and diversity. These differences may be related to various consumer, disease, and nutritional factors. These microarchaeological findings and microbial characteristics may be attributed to different microbial responses to specific consumer habits. Hence, different consumption habits, patterns, and disease profiles lead to different compositions of the generated wastes and thus leave different microbial traces. **This statement includes results and conclusions and does not really fit in the introduction.**

Site (İnönü Cave) and Samples

İnönü Cave is a cave of volcanic origin located in the Cambu geological formation. This volcanic cave, located on the Western Black Sea coast of Turkiye, developed in several stages (Fig 1)[5]
Fig 3: Number of Identified Specimens (%)[(17)]

Why is there a change of reference style (superscript)?

Bioinformatics analysis identified the presence of a wide range of taxa, including Cyanobacteria, the Acidobacteriota phylum, the Actinobacteriota phylum, Bacteroidota, Chloroflexi, Firmicutes, Myxococcota, Proteobacteria, Cyanobacteria, and Nitrospirota species (Tabel 2). **Typo: Table**

Material and Method **(Rather use the plural Materials and Methods)**

Assoc analyzed the defined fillings analogically. Prof. Dr. Hamza Ekmen and Assoc. Prof. Dr. F. Gülden Ekmen and confirmed with 14 C analyses, were taken with sterile materials (Eppendorfs, falcon tubes, steel spatulas, steel collecting spoons) by pharmaceutical microbiologist Assoc. Prof. Dr. Sukran Ozturk

from the Faculty of Pharmacy. **(Paraphrase these sentences, the structure is not correct)** The materials were sterilized in an autoclave (brand Nüve) at 121° C for 15 minutes in the Pharmaceutical Microbiology Laboratory of the Beun Faculty of Pharmacy and transported to the excavation site (Figures **3**-4). Detailed information is shown in Table 1. **(I don't hink the NISP Figure 3 is relevant here)**

PCR and Bioinformatics

Small 16S gene region primers (16SV3-F/16SV3-R) were used for the qPCR test to see if PCR inhibition happened [15]. **Again superscript.**

Data availability
Our data falls under the list of data types that must be deposited with you. We provide the relevant accession numbers, and the name of the database in which your data is stored is "The datasets generated and/or analyzed during the current study are available in the NCBI BioProject repository, BioProcecj ID: PRJNA1134133."
Omit the first part, this is not necessary to mention and just state: ""The datasets generated and/or analyzed during the current study are available in the NCBI BioProject repository, BioProcecj ID: PRJNA1134133."

Results

We have found information supporting our study on these taxa (Fig 6a,b,c,d). **The figures are not supporting this statement but your own results. Leave this sentence out. You are discussing your results in a later part of the results anyways.**

The MN NUCLEOSPIN Soil DNA Extraction Kit was chosen for its proven efficiency in isolating ancient DNA (aDNA) from complex soil matrices, which often contain high levels of PCR inhibitors such as humic acids. This kit's robust inhibitor removal technology ensures higher purity and yield of DNA, which is critical for downstream applications in ancient microbiome studies. Furthermore, its dual-buffer system (SL1 and SL2) optimized lysis efficiency across varied soil types, enhancing DNA recovery from both Gram-positive and Gram-negative bacteria (Fig 6a,b,c,d).
This is a repetition form the Methods section. Shorten or leave out.
Following this paragraph there is an icon of a file named "aDNA_silva_krona.html" in the text. This seems to be a mistake.

The Actinobacteriota phylum, or Actinobacteria, is a diverse group of bacteria found in various environments, including soil, water, and plants. They are characterized by their high G+C content in their genomic DNA and filamentous growth pattern. Actinobacteria are known for their ability to produce a wide range of bioactive compounds, such as antibiotics, antifungals, and antitumor agents. This has led to extensive research into their potential applications in medicine and biotechnology. Additionally, Actinobacteria play essential roles in soil

ecosystems, involving nutrient cycling and organic matter degradation (51).
Another identified bacterial taxon is Bacteroidota. They are commonly found in aquatic environments, soil, and the guts of animals, including humans. Bacteroidota bacteria are essential in breaking down complex organic matter, such as polysaccharides, and play a vital role in the carbon cycle (25).
These detailed descriptions of the phyla seems a bit repetitive as well. Shorten and focus on your results.

Lactobacillus species produce fermented foods such as yogurt and cheese (27, 28). Bacillus species are known for making antibiotics and enzymes, while Clostridium species are essential in producing biogas and other industrial products. Lactobacillus species produce fermented foods such as yogurt and cheese, while Staphylococcus aureus can cause human infections (27, 28).
Repetitive.

Note: The quality of the figures, even after downloading them, is so bad that they could not be evaluated by us.

Conclusio:
This revision added substantial value to the manuscript and after addressing our aforementioned comments, we recommend publication.
