# Supplementary material for: Decoding past microbial life and antibiotic resistance in İnonü Cave’s archaeological soil
Source: PLoS One. 2025 Jul 31;20(7):e0326358. doi: 10.1371/journal.pone.0326358 (PMC12312911; doi:10.1371/journal.pone.0326358)
Supplement: Attachment — Submitted filename: renamed_ba4a1.docx [file pone.0326358.s005.docx]

**Review of “Decoding Past Microbial Life and Antibiotic Resistance in İnonü Cave’s Archaeological Soil”**

In this paper, the authors describe the identification of resistance genes through metabarcoding from archaeological sediments taken from Inönü Cave.

The short title “Archaeology Microbiology Collaboration: soil microbiota and antibiotic resistance genes” might not be the best choice, as “Archaeology Microbiology Collaboration” sounds improper. Consider renaming it (e.g. to “Integrating Archaeology and Microbiology: Insights into Ancient Resistance Genes”)

“*1 These authors contributed equally to this work.”, and ”‘#2 These authors also contributed equally to this work.” There is no necessity to mention this, as there is only a single person listed as the main author and one person as the second author. This should be removed.

**Literature Context**

The manuscript significantly lacks references to support its statements. While the introduction briefly mentions previous studies, it fails to provide a clear, structured overview of the field. At minimum, each claim and mention of prior research should be substantiated with at least one reference. This is a fundamental scientific standard, and addressing this would substantially improve the paper’s quality.

**Language and Clarity**

The language used throughout the manuscript does not meet scientific standards. Phrases like “it is understood,” “it was revealed,” and “it can be said” are vague and unscientific. Additionally, some descriptions, such as “Each sample was tried in duplicate,” are phrased in an unusual way. The manuscript needs a thorough review for grammar, clarity, and precise use of terminology.

Furthermore, the written language does not live up to scientific standards and should be checked for correctness, clarity, and use of the correct and scientific words (e.g., Each sample was tried in duplicate). Be careful with statements such as “it is understood”, “it was revealed”, “it can be said”, as they are highly unspecific.

**Methodology**

The authors mention using a database for bioinformatics but fail to explain the reason for choosing that one and add a reference. Details about the tools, versions, and statistical methods used are also lacking. This is critical information for reproducibility. Furthermore, the exact sampling procedure of the sediment was not described (Who are the mentioned experts? What do they define as “sterile conditions”? This requires specification and references). Moreover, the authentication of the obtained ancient DNA is unclear. How did they assess the authenticity of the results (i.e. if the DNA is ancient as opposed to contamination? Did they conduct contamination tests? Were negative controls included during the wet lab process and the sampling (control samples from non-archeological sediments?). The authors should address these topics.

There is a lack of explanations and references for almost every methodological decision that was made. For instance, it is unclear why "MN NUCLEOSPIN Soil DNA Extraction Kit" was selected, whether it is suitable for aDNA extraction or commonly used, or whether it is the author’s decision to use it for a specific reason. The same applies to other laboratory and bioinformatic tools that were used, which require explanations and/or references.

**Data Availability**

The data availability statement is poorly written and contains typographical errors, such as “Our data falls under the list of data types which must be deposited you.” This section needs a complete rewrite to meet publication standards.

**Results**

The results section is overly simplistic and presented in a textbook-like manner. The descriptions of bacterial taxa belong more to a background section than the results. The quantitative and qualitative data are insufficiently detailed—tables and comprehensive descriptions of findings are necessary.

In the text, the authors mention that they identified specific taxa (“Bioinformatics analysis identified the presence of a wide range of taxa including…”). However, they did not include the criteria for taxa identification. The data generation must be presented in a reproducible manner throughout the paper (methods and results sections).

**Figures and Tables**

The figures are blurry and not reviewable, even after downloading the .tif files. Figures 4 and 5 lack proper descriptions, making them difficult to interpret. High-resolution images and complete figure legends need to be provided.

Table 2 is written in Turkish, this must be changed.

**Discussion**

The discussion repeats information already covered in the results. For example, the origin of resistance genes and characteristics of bacterial taxa are unnecessarily rehashed. The first paragraph of the discussion also needs paraphrasing, including the repetitive mention of "sulfur."

The manuscript has potential but requires major revisions to be considered for publication. The foundational issues - language, structure, references, and data presentation, generation, and **authentication** - must be addressed before resubmission. With significant improvement, this study could contribute valuable insights into the intersection of archaeology and microbiology.
