# Supplementary material for: Decoding past microbial life and antibiotic resistance in İnonü Cave’s archaeological soil
Source: PLoS One. 2025 Jul 31;20(7):e0326358. doi: 10.1371/journal.pone.0326358 (PMC12312911; doi:10.1371/journal.pone.0326358)
Supplement: Attachment — Submitted filename: Response to Reviewers1.docx [file pone.0326358.s008.docx]

**RESPONSE EDITOR AND REVIEWS**

PONE-D-24-53725

Decoding Past Microbial Life and Antibiotic Resistance in İnonü Cave’s Archaeological Soil

PLOS ONE

Dear Dr. Ozturk,

Thank you for submitting your manuscript to PLOS ONE. After careful consideration, we feel that it has merit but does not fully meet PLOS ONE’s publication criteria as it currently stands. Therefore, we invite you to submit a revised version of the manuscript that addresses the points raised during the review process.

==============================

Your manuscript has now been seen by a referee, whose comments are appended below. You will see from these comments that while the referee finds your work of potential interest, the reviewer has raised substantial concerns that must be addressed. In light of these comments, we cannot accept the manuscript for publication, but would be interested in considering a revised version that addresses these serious concerns.

We hope you will find the referee's comments useful as you decide how to proceed. Should presentation of further data and analysis allow you to address these criticisms, we would be happy to look at a substantially revised manuscript. However, please bear in mind that we will be reluctant to approach the referees again in the absence of major revisions.

==============================

Please submit your revised manuscript by Mar 05 2025 11:59PM. If you will need more time than this to complete your revisions, please reply to this message or contact the journal office at [plosone@plos.org](mailto:plosone@plos.org). When you're ready to submit your revision, log on to https://www.editorialmanager.com/pone/ and select the 'Submissions Needing Revision' folder to locate your manuscript file.

Please include the following items when submitting your revised manuscript:

- A rebuttal letter that responds to each point raised by the academic editor and reviewer(s). You should upload this letter as a separate file labeled 'Response to Reviewers'.
- A marked-up copy of your manuscript that highlights changes made to the original version. You should upload this as a separate file labeled 'Revised Manuscript with Track Changes'.
- An unmarked version of your revised paper without tracked changes. You should upload this as a separate file labeled 'Manuscript'.

If you would like to make changes to your financial disclosure, please include your updated statement in your cover letter. Guidelines for resubmitting your figure files are available below the reviewer comments at the end of this letter.

If applicable, we recommend that you deposit your laboratory protocols in protocols.io to enhance the reproducibility of your results. Protocols.io assigns your protocol its own identifier (DOI) so that it can be cited independently in the future. For instructions see: [https://journals.plos.org/plosone/s/submission-guidelines#loc-laboratory-protocols](https://journals.plos.org/plosone/s/submission-guidelines#loc-laboratory-protocols). Additionally, PLOS ONE offers an option for publishing peer-reviewed Lab Protocol articles, which describe protocols hosted on protocols.io. Read more information on sharing protocols at <https://plos.org/protocols?utm_medium=editorial-email&utm_source=authorletters&utm_campaign=protocols>.

We look forward to receiving your revised manuscript.

Kind regards,

Peter F. Biehl, PhD
Academic Editor
PLOS ONE

**Journal Requirements:**

When submitting your revision, we need you to address these additional requirements.

1. Please ensure that your manuscript meets PLOS ONE's style requirements, including those for file naming. The PLOS ONE style templates can be found at 
https://journals.plos.org/plosone/s/file?id=wjVg/PLOSOne_formatting_sample_main_body.pdf and 
https://journals.plos.org/plosone/s/file?id=ba62/PLOSOne_formatting_sample_title_authors_affiliations.pdf

 Response

- It’s revised.

2. PLOS ONE now requires that authors provide the original uncropped and unadjusted images underlying all blot or gel results reported in a submission’s figures or Supporting Information files. This policy and the journal’s other requirements for blot/gel reporting and figure preparation are described in detail at https://journals.plos.org/plosone/s/figures#loc-blot-and-gel-reporting-requirements and https://journals.plos.org/plosone/s/figures#loc-preparing-figures-from-image-files. When you submit your revised manuscript, please ensure that your figures adhere fully to these guidelines and provide the original underlying images for all blot or gel data reported in your submission. See the following link for instructions on providing the original image data: https://journals.plos.org/plosone/s/figures#loc-original-images-for-blots-and-gels.
   In your cover letter, please note whether your blot/gel image data are in Supporting Information or posted at a public data repository, provide the repository URL if relevant, and provide specific details as to which raw blot/gel images, if any, are not available. Email us at plosone@plos.org if you have any questions.

Response

-It’s added

3. PLOS requires an ORCID iD for the corresponding author in Editorial Manager on papers submitted after December 6th, 2016. Please ensure that you have an ORCID iD and that it is validated in Editorial Manager. To do this, go to ‘Update my Information’ (in the upper left-hand corner of the main menu), and click on the Fetch/Validate link next to the ORCID field. This will take you to the ORCID site and allow you to create a new iD or authenticate a pre-existing iD in Editorial Manager.

 Response

- It’s added.

4. In your manuscript, please provide additional information regarding the specimens used in your study. Ensure that you have reported human remain specimen numbers and complete repository information, including museum name and geographic location.

If permits were required, please ensure that you have provided details for all permits that were obtained, including the full name of the issuing authority, and add the following statement:

'All necessary permits were obtained for the described study, which complied with all relevant regulations.'

If no permits were required, please include the following statement:

'No permits were required for the described study, which complied with all relevant regulations.'

For more information on PLOS ONE's requirements for paleontology and archeology research, see https://journals.plos.org/plosone/s/submission-guidelines#loc-paleontology-and-archaeology-research.

 Response

- The necessary information has been added.

- The excavations of İnönü Cave are carried out under the licence granted by the Ministry of Culture and Tourism of the Republic of Turkiye to one of the authors, Hamza Ekmen. All necessary permits were obtained for the described study, which complied with all relevant regulations. The licence granted covers all scientific studies related to İnönü Cave.

All primary archaeological artefacts are stored at the Zonguldak Provincial Karadeniz Ereğli Archaeological Museum and secondary artefacts that require laboratory work are stored at the Zonguldak Bülent Ecevit University Archaeology Department Laboratory in accordance with the relevant licence.

5. In your Methods section, please provide additional information regarding the permits you obtained for the work. Please ensure you have included the full name of the authority that approved the field site access and, if no permits were required, a brief statement explaining why.

Response:

- You can see this link.

<https://kvmgm.ktb.gov.tr/TR-393463/2024-yili-kazi-ve-yuzey-arastirma-faaliyetleri.html>

Authorization institution: Ministry of Culture of the Republic of Turkiye. You can check the list opened via the link above. Please click on ([2024 Yılında Bakanlığımıza Bağlı Müze Müdürlükleri Tarafından Gerçekleştirilen Müze Kazıları](https://kvmgm.ktb.gov.tr/Eklenti/130844,2024yilindabakanligimizabaglimuzemudurlukleritarafindangerceklestirilenmuzekazilaripdf.pdf?0))

6. Thank you for stating the following financial disclosure: 
ZBEUN Scientific Research Coordinator (BAP) number 2021-91149634-02.

Please state what role the funders took in the study.  If the funders had no role, please state: ""The funders had no role in study design, data collection and analysis, decision to publish, or preparation of the manuscript."" 
If this statement is not correct you must amend it as needed. 
Please include this amended Role of Funder statement in your cover letter; we will change the online submission form on your behalf.

 Response

-The funders had no role in study design, data collection and analysis, decision to publish, or preparation of the manuscript."

7. Thank you for stating the following in the Acknowledgments Section of your manuscript: 
The opinions, findings, and conclusions or recommendations expressed in this material are
solely the responsibility of the authors and do not necessarily represent the official views of the funding sources.
This project was made with the support of ZBEUN BAP number 2021-91149634-02. We
Thanked you ZBEUN Scientific Research Coordinator.
We present this article to the Great Leader Mustafa Kemal Atatürk and all our martyrs on the
occasion of the 100th anniversary of Turkiye Republic.

We note that you have provided funding information that is not currently declared in your Funding Statement. However, funding information should not appear in the Acknowledgments section or other areas of your manuscript. We will only publish funding information present in the Funding Statement section of the online submission form. 
Please remove any funding-related text from the manuscript and let us know how you would like to update your Funding Statement. Currently, your Funding Statement reads as follows: 
ZBEUN Scientific Research Coordinator (BAP) number 2021-91149634-02.

Please include your amended statements within your cover letter; we will change the online submission form on your behalf.

 Response

-It’s revised

8. We note that your Data Availability Statement is currently as follows: All relevant data are within the manuscript and its Supporting Information files.

Please confirm at this time whether or not your submission contains all raw data required to replicate the results of your study. Authors must share the “minimal data set” for their submission. PLOS defines the minimal data set to consist of the data required to replicate all study findings reported in the article, as well as related metadata and methods (https://journals.plos.org/plosone/s/data-availability#loc-minimal-data-set-definition).

For example, authors should submit the following data:

- The values behind the means, standard deviations and other measures reported;
- The values used to build graphs;
- The points extracted from images for analysis.

Authors do not need to submit their entire data set if only a portion of the data was used in the reported study.

If your submission does not contain these data, please either upload them as Supporting Information files or deposit them to a stable, public repository and provide us with the relevant URLs, DOIs, or accession numbers. For a list of recommended repositories, please see https://journals.plos.org/plosone/s/recommended-repositories.

If there are ethical or legal restrictions on sharing a de-identified data set, please explain them in detail (e.g., data contain potentially sensitive information, data are owned by a third-party organization, etc.) and who has imposed them (e.g., an ethics committee). Please also provide contact information for a data access committee, ethics committee, or other institutional body to which data requests may be sent. If data are owned by a third party, please indicate how others may request data access.

Response

-It’s revised

9. Please amend your list of authors on the manuscript to ensure that each author is linked to an affiliation. Authors’ affiliations should reflect the institution where the work was done (if authors moved subsequently, you can also list the new affiliation stating “current affiliation:….” as necessary)

Response

-Its revised

10. Please include a separate caption for each figure in your manuscript.

Additional Editor Comments :

Your manuscript has now been seen by a referee, whose comments are appended below. You will see from these comments that while the referee finds your work of potential interest, the reviewer has raised substantial concerns that must be addressed. In light of these comments, we cannot accept the manuscript for publication, but would be interested in considering a revised version that addresses these serious concerns.

We hope you will find the referee's comments useful as you decide how to proceed. Should presentation of further data and analysis allow you to address these criticisms, we would be happy to look at a substantially revised manuscript. However, please bear in mind that we will be reluctant to approach the referees again in the absence of major revisions.


[Note: HTML markup is below. Please do not edit.]

Reviewers' comments:

Reviewer's Responses to Questions

Response

Dear Editor

First of all, thank you for giving us this opportunity.

I and all the authors are honored that our article can be published in a distinguished journal like Plos One.

This study has been meticulously done and all the analyses that needed to be done within the budget we had were done and verified, and it has been presented as an article that brings together two different disciplines, archaeology and microbiology, which is rare and has potential for development. All your suggestions have been taken into consideration and revisions have been completed so that our article can be published in your journal.

Thank you.

Best regards,

The manuscript has been revised according to the referee suggestions.

**Comments to the Author**

1. Is the manuscript technically sound, and do the data support the conclusions?

The manuscript must describe a technically sound piece of scientific research with data that supports the conclusions. Experiments must have been conducted rigorously, with appropriate controls, replication, and sample sizes. The conclusions must be drawn appropriately based on the data presented.

Reviewer #1: Partly

2. Has the statistical analysis been performed appropriately and rigorously?

Reviewer #1: I Don't Know

3. Have the authors made all data underlying the findings in their manuscript fully available?

The [PLOS Data policy](http://www.plosone.org/static/policies.action#sharing) requires authors to make all data underlying the findings described in their manuscript fully available without restriction, with rare exception (please refer to the Data Availability Statement in the manuscript PDF file). The data should be provided as part of the manuscript or its supporting information, or deposited to a public repository. For example, in addition to summary statistics, the data points behind means, medians and variance measures should be available. If there are restrictions on publicly sharing data—e.g. participant privacy or use of data from a third party—those must be specified.

Reviewer #1: Yes

4. Is the manuscript presented in an intelligible fashion and written in standard English?

PLOS ONE does not copyedit accepted manuscripts, so the language in submitted articles must be clear, correct, and unambiguous. Any typographical or grammatical errors should be corrected at revision, so please note any specific errors here.

Reviewer #1: No

5. Review Comments to the Author

Please use the space provided to explain your answers to the questions above. You may also include additional comments for the author, including concerns about dual publication, research ethics, or publication ethics. (Please upload your review as an attachment if it exceeds 20,000 characters)

Reviewer #1: Ad 1) The manuscript is partly technically sound as the authors do not comprehensively explain the methodologies and fail to explain why they chose specific tools.

Response 1)

**1. Soil DNA Sampling and Extraction Section**

"The MN NUCLEOSPIN Soil DNA Extraction Kit was chosen for its proven efficiency in isolating ancient DNA (aDNA) from complex soil matrices, which often contain high levels of PCR inhibitors such as humic acids. This kit's robust inhibitor removal technology ensures higher purity and yield of DNA, which is critical for downstream applications in ancient microbiome studies. Furthermore, its dual-buffer system (SL1 and SL2) was utilized to optimize lysis efficiency across varied soil types, enhancing DNA recovery from both Gram-positive and Gram-negative bacteria."

**2. PCR and Bioinformatics Section**

"The 16SV3-F/16SV3-R primers were selected for their specificity in targeting the hypervariable V3 region of the 16S rRNA gene, providing high-resolution taxonomic identification of bacterial communities. This region is well-documented in ancient DNA studies due to its ability to generate reliable phylogenetic data even from fragmented DNA. For antibiotic resistance gene detection, primer pairs targeting blaTEM, tetA, intl1, and OXA58 were chosen based on their widespread validation in environmental and archaeological microbiology. These primers have demonstrated high specificity and sensitivity in detecting resistance genes from degraded DNA templates, ensuring accurate representation of ancient resistomes."

**3. Sequencing Method Justification**

"Illumina iSeq (2x150 paired-end) sequencing was selected due to its high-throughput capabilities and exceptional accuracy in short-read sequencing, which is essential for ancient DNA studies. The platform's ability to produce large volumes of high-quality data at a lower cost makes it ideal for comprehensive metagenomic analyses. Additionally, the 150 bp paired-end reads are particularly suited for amplifying the short, fragmented nature of ancient DNA, ensuring maximal recovery of informative sequences."

**4. Bioinformatics Pipeline Justification**

"OBITOOLS was employed due to its specialized capabilities in metabarcoding analyses, offering a comprehensive suite of tools for sequence filtering, taxonomic assignment, and error correction, all of which are critical for handling ancient DNA datasets prone to degradation and contamination. The SILVA database was utilized for taxonomic identification because of its extensive, curated repository of ribosomal RNA sequences, providing high-confidence taxonomic resolution essential for accurate characterization of ancient microbial communities. Quality control was rigorously enforced using FastQC, with a stringent Phred score threshold of 30 to ensure the reliability of sequence data, minimizing errors and artifacts common in ancient DNA sequencing."

2) There is no description on the statistical methods included in the paper, also no settings and threshold of the employed bioinformatics tools.

**Response2)**

**Statistical Analysis**

To assess the diversity and composition of microbial communities across different archaeological layers, we employed several statistical analyses. Alpha diversity metrics, including Shannon and Simpson indices, were calculated using QIIME2 to evaluate species richness and evenness within samples. Beta diversity was assessed using Bray-Curtis dissimilarity and visualized through Principal Coordinates Analysis (PCoA) to compare microbial community structures between samples from different time periods. Differential abundance analysis was conducted using the DESeq2 package in R to identify significant variations in taxa abundance across the archaeological layers. P-values were adjusted for multiple comparisons using the Benjamini-Hochberg procedure, with a significance threshold set at p < 0.05. For the detection of antibiotic resistance genes, quantitative PCR (qPCR) data were analyzed using standard curve methods to quantify gene copy numbers. Statistical significance of differences in resistance gene abundance across time periods was evaluated using one-way ANOVA, followed by post hoc Tukey’s tests where applicable.

**Bioinformatics Settings and Thresholds**

Sequences were merged using the illuminapairedend tool with default overlap settings. Reads with ambiguous bases or shorter than 150 bp were discarded to ensure data quality. Chimeric sequences were identified and removed using the uchime-denovo algorithm in VSEARCH. Taxonomic assignments were made with a confidence threshold of 80% using the SILVA 138 database. Rarefaction was performed to a depth of 90,000 reads per sample to standardize sequencing effort across all samples.

Response3)
3) Yes, it is deposited.

4) The language requires major revision.

Response 4- The language is proofed

By integrating these sections into the manuscript, we comprehensively address the reviewers' concerns regarding the methodologies and the rationale behind tool selection.

6. PLOS authors have the option to publish the peer review history of their article ([what does this mean?](https://journals.plos.org/plosone/s/editorial-and-peer-review-process#loc-peer-review-history)). If published, this will include your full peer review and any attached files.


If you choose “no”, your identity will remain anonymous but your review may still be made public.


**Do you want your identity to be public for this peer review?** For information about this choice, including consent withdrawal, please see our [Privacy Policy](https://www.plos.org/privacy-policy).

Reviewer #1: No

[NOTE: If reviewer comments were submitted as an attachment file, they will be attached to this email and accessible via the submission site. Please log into your account, locate the manuscript record, and check for the action link "View Attachments". If this link does not appear, there are no attachment files.]

While revising your submission, please upload your figure files to the Preflight Analysis and Conversion Engine (PACE) digital diagnostic tool, <https://pacev2.apexcovantage.com/>. PACE helps ensure that figures meet PLOS requirements. To use PACE, you must first register as a user. Registration is free. Then, login and navigate to the UPLOAD tab, where you will find detailed instructions on how to use the tool. If you encounter any issues or have any questions when using PACE, please email PLOS at [figures@plos.org](mailto:figures@plos.org). Please note that Supporting Information files do not need this step.

 Response

- While revising our submission, has been upload all our figure files to the Preflight Analysis and Conversion Engine (PACE). All figures have been converted to TIFF and PDF formats in the PACE program.
